# Obesity-Related Glomerulosclerosis—How Adiposity Damages the Kidneys

**DOI:** 10.3390/ijms26136247

**Published:** 2025-06-28

**Authors:** Justyna Zbrzeźniak-Suszczewicz, Agata Winiarska, Agnieszka Perkowska-Ptasińska, Tomasz Stompór

**Affiliations:** 1 Department of Nephrology, Hypertension and Internal Medicine, University of Warmia and Mazury in Olsztyn, 10-719 Olsztyn, Poland; j.zbrzezniak@op.pl (J.Z.-S.); agatawiniarska@hotmail.com (A.W.); 2 Department of Pathology, Medical University of Warsaw, 02-091 Warsaw, Poland; agnieszka.perkowska-ptasinska@wum.edu.pl

**Keywords:** obesity, obesity-related glomerulosclerosis (ORG), chronic kidney disease (CKD), proteinuria, albuminuria, focal and segmental glomerulosclerosis (FSGS), hyperfitration, incretine-based therapies, bariatric surgery, weight loss

## Abstract

Obesity, hypertension, and chronic kidney disease (CKD) constitute the deadly trinity of modern threats for populations of both developed and developing countries. These diseases (together with type 2 diabetes) are closely linked in their pathophysiology and result in increasing cardiovascular (CV) morbidity and premature death from CV causes. In this review, we focused on the kidney as the target of obesity-related disorders. Obesity-related glomerulosclerosis (ORG) represents a pattern of renal injury caused solely or predominantly by obesity; usually, it is superimposed on chronic kidney disease (CKD) from other causes, such as diabetic kidney disease, hypertensive kidney disease, type 2 cardiorenal syndrome, primary or secondary glomerulopathies, and others. Adipose tissue contributes to kidney injury in several ways: it releases proinflammatory cytokines and growth factors, leading to podocyte and mesangial cell injury and glomerulosclerosis. In particular, perirenal adipose tissue (PRAT), besides exerting paracrine and endocrine effects on the kidney, modifies its function via compression on renal parenchyma and vessels. The intrinsic ability of the kidneys in obesity to increase the reabsorption of sodium warrants intraglomerular hypertension and hyperfiltration, followed by progressive renal injury. Lifestyle interventions and pharmacological agents, as well as metabolic (bariatric) surgery resulting in weight reduction, may also be beneficial for the kidneys. Using GLP1 receptor agonists (with a special focus on subcutaneous semaglutide and tirzepatide) seems to be the most promising treatment strategy for preventing kidney injury in obese individuals.

## 1. Introduction

Nowadays, obesity is one of the leading worldwide health problems, with prevalence steeply increasing in recent decades. Obesity has a multifactorial etiology and is characterized by the presence of excess fat deposits with adipocyte hyperplasia and hypertrophy, reflected as a body mass index (BMI) greater than 30 kg/m^2^. The prevalence of obesity in the USA among the adult population reached 35% in 2015, with more than 600 million adults obese worldwide, and the prevalence was higher among women than men in most countries [1,2]. The problem will become even more serious: according to the World Obesity Federation report published in 2019, the estimated number of children and adolescents (age 5–19) with obesity will reach 206 million and will increase to 254 million in 2030 worldwide [3]. Moreover, observational studies show that maternal obesity is associated with long and short-term health consequences for offspring, including metabolic disorders (e.g., childhood obesity, insulin resistance and type 2 diabetes, and metabolic syndrome), cardiovascular diseases (stroke and coronary heart disease), hormonal and immune dysregulation, and neurodevelopmental disorders [4]. A large proportion of these individuals will experience obesity-related complications, including obesity-related glomerulopathy (ORG). It is important to mention that in some studies, ORG may be more common in males, possibly due to differences in fat distribution, hormonal factors, and lifestyle behaviors [5]. Mechanisms initiating obesity-related lesions within the kidneys are not fully understood. Animal models of obesity clearly demonstrate that weight loss improves kidney function and reverses histological lesions of ORG—these data suggest a direct link between obesity and advanced renal injury, which might be reversible [6].

In this review, we aimed to discuss the pathophysiological background of kidney injury associated with obesity and perirenal adipose tissue accumulation. We also discussed recent clinical trials performed in obese patients with and without type 2 diabetes, addressing certain renal end-points.

### 1.1. Obesity-Related Glomerulosclerosis (ORG)—Kidney as a Victim of Adipose Tissue

**Figure 1:**
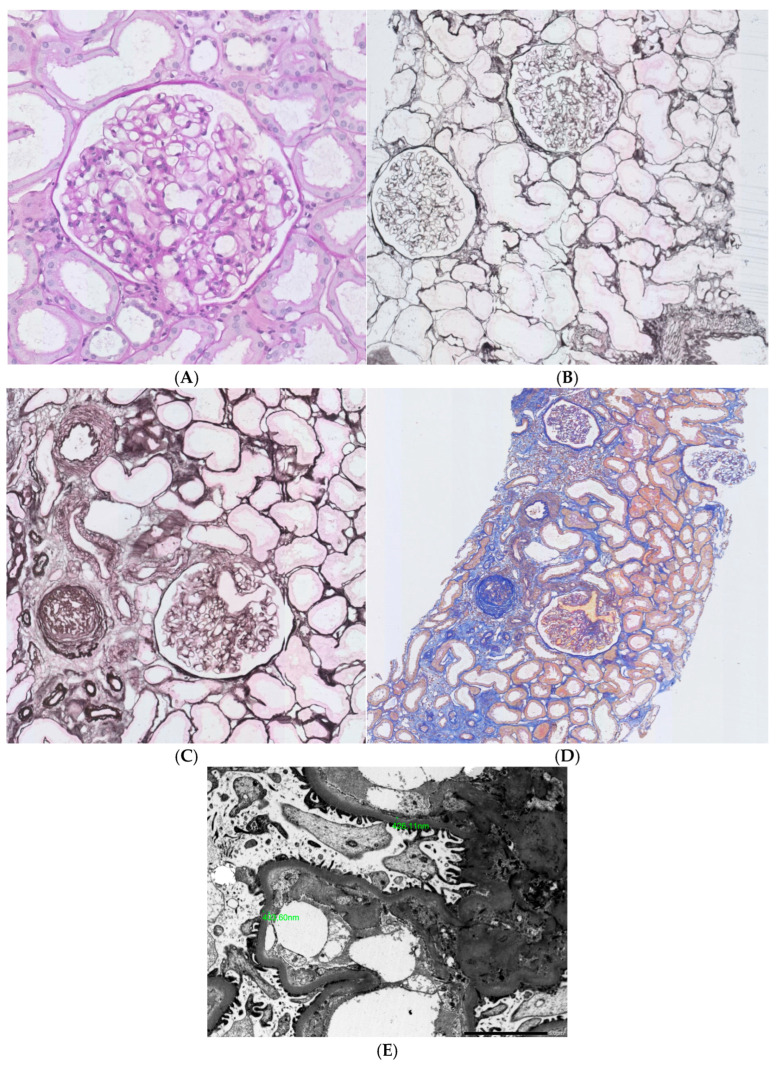
Pathologic lesions typical for obesity-related glomerulosclerosis (**A**). Glomerulomegaly, PAS stain; (**B**). Glomerulomegaly, silver methamine; (**C**). Focal GS, small foci of fibrosis and tubular atrophy, AFOG stain; (**D**). Focal GS, small foci of fibrosis and tubular atrophy, silver methamine; (**E**). ORG, mild thickening of GBM_X2500]. The most characteristic finding is a triad of lesions, encompassing glomerulomegaly and focal segmental glomerulosclerosis on light microscopy, along with normal or only mildly increased thickness of the glomerular basement membrane (GBM) on ultrastructural examination. Occasionally, mild mesangial widening and focal effacement of podocyte foot processes may also be seen.

Obesity seems to be one of the most important (following diabetes) causes of chronic kidney disease (CKD) and is an independent factor contributing to this condition [7]. Based on analysis of the Framingham Health Study cohort, obese participants had a 68% higher risk of reaching CKD stage 3 [8]. Overweight/obesity or central fat distribution were shown to be risk factors for renal impairment even after adjustment for the confounding variables [9]. Kidney biopsy remains the gold standard for the diagnosis of glomerular disease. Not so long ago, pathologic reports describing ORG were limited only to autopsy studies, based on which an association between obesity and glomerulomegaly was established [10]. The evaluation of kidney biopsy samples obtained at Columbia University revealed about a 10-fold increase in the incidence of ORG between the end of the 1980s and 1990s (indications for kidney biopsies were routine for clinical practice). The key criteria for making a diagnosis of ORG were the presence of glomerulomegaly with or without the coexistence of focal segmental glomerulosclerosis (FSGS) and a BMI ≥ 30 kg/m^2^. In the analyzed group, the most common pathological findings were glomerulomegaly and FSGS; the glomerular size (diameter) was, on average, 1.34-fold greater when compared to patients without obesity [5]. Similar data were published by Chen et al., who enrolled 46 patients with obesity in the biopsy study and compared them with 10 non-obese, otherwise healthy controls. The volume of glomeruli was increased, and the podocyte density was decreased in obese patients, with differences statistically significant when compared to controls. Moreover, the severity of proteinuria correlated inversely with podocyte density and number [11]. The clinical presentation of ORG may vary from mild to severe proteinuria with or without renal impairment; commonly, nephrotic range proteinuria is present, but without signs of nephrotic syndrome, such as peripheral edema or low serum albumin. This clinical manifestation is strikingly different from primary FSGS, minimal change disease, membranous nephropathy, and advanced diabetic kidney disease—proteinuric glomerular diseases manifesting with fully symptomatic nephrotic syndrome. The prognosis of ORG is rather good (especially when compared to primary FSGS and if not superimposed on CKD from other causes), and typically, proteinuria remains stable or progresses slowly. It is estimated that between 10 and 33% of patients may progress to advanced stages of CKD and ultimately to end-stage kidney disease [12]. Previously, ORG has been thought to be a consequence of hemodynamic changes alone, but the pattern of injury present in histologic assessment cannot be explained only by disturbed flow. Hyperfiltration secondary to afferent arteriole dilatation (driven by decreased tubuloglomerular feedback secondary to the increased proximal tubule sodium reabsorption and decreased sodium load reaching macula densa) still remains the important mechanism of injury (sometimes even called ‘glomerular barotrauma’). Nevertheless, now it seems obvious that circulating factors (cytokines, hormones, growth factors) synthetized and released from adipose tissue are the culprits of kidney injury. In the histologic evaluation ORG more often presents as perihilar variant FSGS, probably because of increased pressure at the afferent arteriole. Moreover, hyalinosis and mesangial lesions, especially increased cellularity followed by mesangial matrix are present. Podocytes may be detached from the glomerular basement membrane, in non-sclerosed portions of glomeruli swollen podocytes with vacuolated cytoplasms can be seen [13,14,15]. Focal intracellular lipid vacuoles accumulated in proximal tubular epithelial cells and in glomeruli may also be present [16]. The immunofluorescence findings are nonspecific in the area of glomerulosclerosis, mostly consisting of immunoglobulins and complement components (IgG, IgM, C3) [5]. Images of lesions representative for ORG are shown in Figure 1A–E.

In obesity, several angiogenic and proinflammatory adipokines are secreted from adipose tissue (i.a. angiopoietins, vascular endothelial growth factor [VEGF], cathepsins, cystatin C) [5,17]. Berfield et al. evaluated the influence of insulin-like growth factor-1 (IGF-1) on intracellular lipid accumulation in mesangial cells; those cells later transform into the foam cells. They demonstrated that IGF-1 causes accumulation of lipids in mesangial cells decreasing their ability to respond to specific migratory and contractile stimuli [18]. In addition, lipid accumulation in mesangial cells can be promoted by LDL-feedback regulation stimulated by inflammation [19]. Coimbra et al. tracked changes in the kidneys of obese Zucker rats (fa/fa rats) between weeks 6 to 60 following birth. By week 6, the animals developed hyperinsulinemia and hyperlipidemia and after 14 weeks, diabetes. In 18 week old fa/fa rats, FSGS was seen, and in 40 week old rats tubulointerstitial damage and proteinuria were detected. A 1.8-fold increase in glomerular monocyte/macrophage counts in fa/fa rats was observed when compared to controls and an increase in de novo desmin expression in podocytes was demonstrated, which correlated with damage in adjacent tubular cells. Moreover, increase in the number of mitochondria, intracytoplasmic proteins and fat droplets were seen in podocytes [20].

Adipose tissue producing and secreting adipokines is now recognized as an active endocrine tissue. Studies show that the level of adiponectin concentration is low in obesity, and there is an inverse correlation between its serum level and increased cardiovascular mortality [21]. The same correlation is observed in patient with stage 4 and 5 CKD with metabolic syndrome [22]. Ohashi et al. performed subtotal (5/6) nephrectomy in adiponectin-knockout and wild-type mice. In kidney biopsies from adiponectin-knockout mice, podocyte foot process effacement was noticed in electron microscopy of remnant kidneys, and treatment with recombinant adiponectin reversed proteinuria and histopathologic lesions; it also decreased urinary hydrogen peroxide (reflecting the degree of an oxidative stress) [23]. Adiponectin directly influences kidney structure and function, and its reduced concentration is associated with the development of metabolic syndrome, obesity, diabetes type 2 and hyperlipidemia [24,25]. Meyvis et al. in the study conducted in overweight and obese nondiabetic patients noticed inverse correlation between serum adiponectin and albuminuria [26]. Adiponectin has anti-inflammatory properties executed by the suppression of M1 macrophages and promotion of macrophage differentiation into the M2 phenotype. These actions decrease synthesis of proinflammatory cytokines (e.g., TNF-α and Il-6), and their reduced signaling at the level of mesangial and tubular cells, and podocytes [27]. Moreover, the anti-inflammatory effect of adiponectin on mesangial cells can also be exercised by the reduced NF-κB signaling (the master transcription factor that governs the expression of ‘proinflammatory’ genes [28]. Some authors suggest that high serum adiponectin concentration may be a novel and clinically useful biomarker of renal dysfunction [29]. Sharma et al. in their study comprising a cohort of patients at high risk for developing diabetes and kidney disease as well as in the model of adiponectin gene-knockout (Ad(-/-)) mice showed that adiponectin may have renoprotective effects on podocytes, by reducing their apoptosis through activating AMP-activated protein kinase (AMPK) and suppressing oxidative stress [30]. Another important study on an experimental model was conducted by Rutkowski et al.—they suggested that adiponectin may help to maintain the slit diaphragm structure by reducing mitochondrial dysfunction and endoplasmic reticulum stress in podocytes [31]. Adiponectin exerts effect also on mesangial cells by reducing platelet-derived growth factor (PDGF)-induced mesangial cell proliferation by blocking mTOR pathway and decreasing fibronectin and collagen type IV deposition, thus preventing glomerulosclerosis [32]. Leptin is another adipokine produced mainly by white adipose tissue, cleared predominantly by the kidneys—its serum concentration is increased in patients with CKD [33]. Leptin among other effects stimulates oxidative stress causing endothelial dysfunction and platelet aggregation [34,35]. Moreover, leptin can promote glomerulosclerosis and interstitial fibrosis [36]. Another important factor contributing to the development and progression of ORG is inflammation. The influence of inflammation on progression of CKD is crucial; proinflammatory cytokines can exacerbate kidney fibrosis, endothelial dysfunction, and activation of glomerular and endothelial cells [37]. Recent studies demonstrate that visceral adipose tissue is responsible for synthesis of cytokines other than leptin and adiponectin—it can also produce tumor necrosis factor (TNF), transforming growth factor (TGF) or monocyte chemoattractant protein-1 (MCP-1) [38].

Obesity is related to overfeeding and results in insulin resistance. Insulin resistance induces glomerulosclerosis by several pathways. It influences glomerular hemodynamics by means of afferent arteriole dilatation and efferent arteriole constriction. Hyperinsulinemia leads direct podocyte injury and may contribute to podocyte loss, inducing lipotoxicity and activating proinflammatory pathways [39,40]. Excess consumption of fructose—rich products induces systemic blood pressure increase, promotes inflammation and oxidative stress within glomerulus, may induce iron-dependent podocyte damage and mesangial injury [41]. Altogether fructose promotes extracellular matrix accumulation, i.e., glomerulosclerosis. Advanced glycation end products—receptor for advanced glycation end products (AGE-RAGE) signaling is well-recognized pathway of tissue injury in obesity (and may result from glycation of endogenous proteins and intake of AGE-modified products) and plays important role in development of glomerular injury in diabetes and beyond [42,43].

To the best of our knowledge, certain gene polymorphisms or gene expression were not studied specifically regarding ORG and differentiating genetic background of ORG vs. obesity in general. Nevertheless, since the expression and activity of several adipokines and cytokines depends on certain gene expression, such a difference cannot be excluded [44].

### 1.2. Hyperfiltration—Phenomenon of ORG

Glomerular hyperfiltration represents a response to increased metabolic needs in obesity. There is a correlation between BMI and podocyte and glomerular hypertrophy in obese patients without renal disease [45]. Some experimental studies were conducted to evaluate response of the podocytes to mechanical stress generated by glomerular pulsation (as a result of an afferent arteriole dilatation). Endlich et al. in their study cultured differentiated mouse podocytes on stretchable silicone membranes and analyzed their structure following mechanical stress application. This experiments showed proof that the podocyte cytoskeleton becomes reorganized as an adaptive response to pulsatile strain. This response promotes podocyte swelling and widening of foot processes that may result in podocyte detachment and the direct adherence of glomerular tuft to Bowman’s capsule (glomerular tuft adhesion) [46]. Only a few publications are available that directly address glomerular filtration in obese patients. In most of them, GFR without correction to body surface was higher when compared to controls (i.e., those with normal body weight) [47]. Chagnac et al. analyzed clearances of several substances to estimate glomerular filtration in nondiabetic patients with severe obesity. Compared to healthy controls, glomerular filtration rate (GFR) and renal plasma flow (RPF) in the studied group were increased by 51 and 31%, respectively. Authors concluded that renal vasodilatation contributing to hyperfiltration involves mainly or solely the afferent arteriole [14]. In fact, several factors may induce hyperfiltration, but afferent/efferent arteriole seems crucial. Hyperfiltration is more likely to occur in patients who experience a low nephron number, have acquired nephron loss, have an AGE-modified structure of arterioles, and those who eat high-protein diet. All substances that modify afferent/efferent arteriole balance that pose vasoconstrictive or vasodilating potential may be involved in development of hyperfiltration and include (among others): renin, angiotensin II, aldosterone, endothelin, nitric oxide, adenosine, osmotic agents in urine, etc. Hyperfiltration may also depend on hydration status and diuretic use. All these factors may contribute to hyperfiltration in obese patients, who otherwise are predisposed to this phenomenon [48]. In addition, high GFR in studied group may be caused by transcapillary hydraulic pressure gradient, mainly associated with increased arterial pressure in capillaries due to dilated afferent arteriole [14]. These findings suggest that systemic hypertension may play a role in pathogenesis of hyperfiltration. The renin–angiotensin–aldosterone system (RAAS) is overactivated probably as a result of increased number of adipocytes that synthesize RAAS components [49]. The ability of angiotensin II to increase sodium reabsorption in proximal tubules by stimulating the Na^+^-H^+^ changer, Na^+^-K^+^-ATPase, and Na^+^ channel (ENaC) is well recognized [50]. In addition, the renal sympathetic nervous system (RSNS) overactivated in obesity and also promotes sodium reabsorption. Leptin and adiponectin levels are known factors to activate RSNS [51,52]. Another theory explaining hemodynamic changes in ORG is the ‘tubular’ hypothesis. Increases filtered sodium reabsorption in proximal tubules results decreased concentration of sodium in the filtrate reaching the macula densa. activating a negative tubule–glomerular feedback loop and vasodilation of the afferent arteriole [53]. An increased filtration fraction as a consequence of obesity-related hyperfiltration causes hemoconcentration in postglomerular vessels and increased oncotic pressure in peritubular capillaries that is expected to promote proximal tubular sodium reabsorption [54]. Glomerular hypertension is associated with increased capillary wall stress, which may cause hyperfiltration and structural abnormalities such as glomerulomegaly or basement membrane thickening [55]. Fluid flow shear stress exerted on podocytes leads to their detachment and activates mediators that promote glomerular injury and sclerosis (angiotensin II, angiotensin II type 1 receptor, transforming growth factor β—TGB β, transforming growth factor β receptor, and phospholipase D). Those factors may promote apoptosis, decreased adhesion, and detachment of podocytes, as well as their hypertrophy [56,57,58].

### 1.3. The Potential Role of Adipose Tissue Surrounding the Kidneys in Development of ORG

Several recent papers show that obesity and increase in mass of adipose tissue is correlated with metabolically active peri-renal adipose tissue (PRAT). PRAT is a part of visceral adipose tissue (mixed brown and white) surrounding the kidney, in the past thought to play a role as a mechanical support for this organ, now known to influence kidney and cardiovascular system function. The association between PRAT, glucose concentration, and metabolic syndrome has been discovered; PRAT contributes to the increased cardiovascular risk, insulin resistance and proatherogenic dyslipidemia [59,60]. The association between visceral adipose tissue (VAT) and renal and cardiovascular system disorders is recognized for decades, but now it seems that such association is even stronger for PRAT. PRAT synthesizing and releasing adipocytes functions as an endocrine and paracrine tissue influencing lipid and carbohydrate metabolism and governing inflammatory processes [61]. PRAT can release the potent proinflammatory mediators (including IL-1β, IL6, TNF-α, leptin, lipocalin, vimentin, and resistin), which may promote injury of a kidney tissue on a paracrine and endocrine manner and may also contribute to damage of remote organs, such as the heart and liver [62,63]. PRAT is also a source of free fatty acid supply to the kidney, thus promoting lipotoxicity, one of the key mechanisms of kidney injury in diabetic and non-diabetic kidney disease. Free fatty acids released by PRAT may impair endothelial function [64]. Inflammatory agents can affect renal function in a paracrine way through leptin, interleukin-1β or TNF [65]. Autonomic nervous system abundantly innervates PRAT, and in obesity its overactivation is common. Autonomic nerve stimulation enhanced by PRAT may contribute to hypertension [66]. Cao et al. performed a study in the Sprague Dawley rats that were randomized into three groups: control rats receiving sham operation (implantation of carotid baroreceptor stimulating device, without stimulation), obese rats receiving sham operation (like previous group), and obese rats receiving carotid baroreceptor stimulation device. The result was reduced metabolic disorders and insulin resistance (including decrease in PRAT mass and adipocyte hypertrophy) in obese rats receiving stimulation of baroreceptors and, thus, inhibiting the sympathetic nervous system [67]. PRAT has a unique feature differentiating it from VAT in other locations, namely it is surrounded by a tight fibrous membrane—renal fascia. This anatomical condition implicates the special feature of PRAT—its accumulation may have a direct mechanical impact on the kidney causing compression of renal parenchyma and vessels and in consequence impact on renal hemodynamics, GFR, sodium reabsorption and blood pressure. Excess adipose tissue localized in the region of renal sinus (also belonging to PRAT) may exert direct pressure on renal vein and lymphatic vessels, thus increasing intrarenal pressure and impairing GFR [62]. Chen et al. evaluated the association between PRAT measured using magnetic resonance imaging (MRI) and several parameters characterizing renal hemodynamics. They found that patients in the highest tertile of PRAT were characterized by the lowest values GFR, renal plasma flow and glomerular hydrostatic pressure as compared to patients in the lowest tertile of PRAT. On the contrary, in patients with the highest values of PRAT, the highest values of renal vascular resistance and afferent arteriole resistance were found [68]. Several trials, both cross-sectional and prospective, have demonstrated that PRAT thickness (evaluated usually by using ultrasound and less frequently by MRI or computed tomography (CT)) correlates with albuminuria and lowered eGFR and predicts the risk of developing CKD, mostly in patients with diabetes and/or obesity but also those with hypertension [68,69]. Certain thresholds of PRAT thickness that differentiate low vs. high risk patients were defined by several authors (these threshold values as well as PRAT thickness considered as ‘normal’ differ between studies due to different techniques of assessment). Hu et al. published a study of special interest for the nephrologist, apparently not directly related to the topic of this review, but very important for documenting the importance of PRAT in the development of renal injury. Namely, they measured perirenal fat thickness (PFT) in patients with biopsy-proven IgA nephropathy (IgAN) and demonstrated that high PFT discriminates the high risk of progression to renal events (doubling of baseline serum creatinine or end-stage renal disease) in IgAN patients. This study has been performed in the group of slim patients with very low prevalence of diabetes and still PFT was the key independent factor of progression to advanced stages of CKD [70].

**Figure 2:**
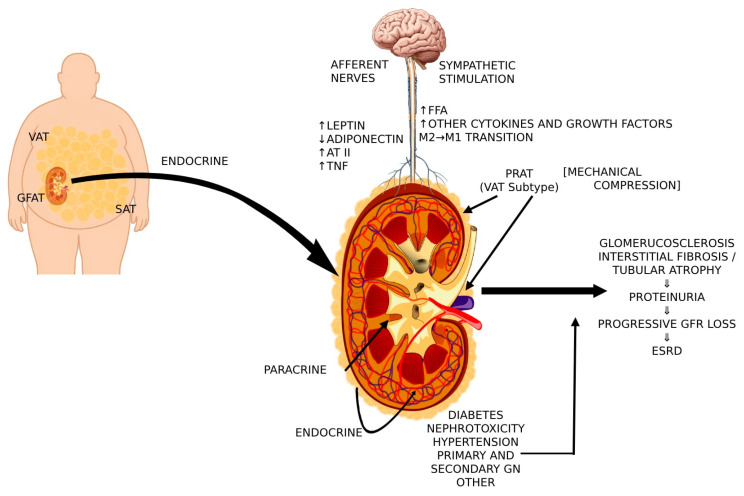
Cross-talk between PRAT, VAT, central nervous system, and sympathetic activity that contributes to renal injury in overweight/obesity [VAT, visceral adipose tissue; GFAT, gluteal-femoral adipose tissue; SAT, subcutaneous adipose tissue; AT II, angiotensin II; TNF, tumor necrosis factor; FFA, free fatty acids; PRAT, perirenal adipose tissue].

The contribution of various adipose tissue-related factors in kidney injury in overweight/obesity are summarized on Figure 2.

## 2. Treatment of ORG

### 2.1. Weight Loss

Since obesity is a trigger of ORG weight loss should be the first therapeutic option in preventing obesity-related organ damage. Even though large, randomized control trials are lacking, data from observational studies prove that weight loss is associated with significant reduction in proteinuria and stabilization of eGFR. Navaneethan et al. analyzed 13 trials, including 11 observational and 2 randomized control trials, describing nonsurgical interventions (diet, exercise and/or medications promoting weight loss; 174 patients in total) and bariatric surgery (including gastric bypass, gastroplasty, and biliopancreatic diversion; 332 patients in total) in patients with preexisting CKD. Trials included in this analysis comprised of obese patients with different types of CKD, i.e., diabetic kidney disease, hypertensive nephrosclerosis, glomerulonephritis, ORG, or proteinuria of unknown origin (in most cases CKD diagnoses were established based on clinical criteria, without biopsy confirmation). Weight reduction in the first group (nonsurgical intervention) resulted in the reduction in proteinuria and decrease in blood pressure and serum cholesterol concentration but without a statistically significant change in eGFR. Bariatric surgery in patients with hyperfiltration leads to normalization of eGFR and similarly to a previous group—to the reduction in proteinuria and better control of hypertension [71]. One can suspect that this effect was due to greater and more sustained weight loss following surgery as compared to non-surgical interventions. With regard to this treatment approach, it is worth mentioning the Swedish Obese Subjects (SOS) study results. It was a non-randomized, matched, prospective intervention study with 4047 participants (2010 patients were included in the bariatric surgery group, and 2037 patients were enrolled in the matched control group). The long-term incidence of ESRD was reduced by more than 70% and the incidence of CKD4/ESRD by 65% in the surgery group compared to the control population receiving non-surgical usual care [72].

The primary goal of obesity treatment is not to reduce proteinuria or postpone progression of CKD to end-stage renal failure. First of all, we want to keep our obese patients alive and bariatric surgery allows to do so. Conley et al. published results of their retrospective cohort study that investigated the association between surgical treatment of obesity and risk of mortality for up to 5 years in patients with CKD G3-5. CKD patients who underwent bariatric surgery (*n* = 802) were matched to controls (*n* = 4933). Results were spectacular—bariatric surgery was associated with a 79% lower 5-year mortality risk as compared to matched controls. Optimistic message from this trial was unfortunately not confirmed by the Cochrane Library analysis. It included 17 RCTs enrolling 988 overweight or obese adults with CKD. Weight loss was achieved by lifestyle modification (exercise, diet), appetite suppressing agents or bariatric surgery. Weight loss appeared to impact significantly only on the reduction in LDL cholesterol while effects on eGFR, proteinuria, SBP, DBP, blood glucose, HbA1C remained uncertain [73]. Unfortunately, none of the trials evaluated risk of cardiovascular events or death. Nevertheless, lifestyle modifications as the cornerstone of therapy cannot be abandoned. Healthy lifestyle should be applied as an upfront intervention also as the key prevention strategy and treatment of ORG, nowadays preferably followed by incretin-based therapies and bariatric surgery. This sequence of interventions is based mainly on safety reasons since bariatric surgery caries 17% risk of complication and 7% risk of reoperation [74].

Weight loss may be especially beneficial for patient with co-occurrence of obesity and diabetes because many studies proved that it may induce the remission of T2DM (losing 15 kg lead to remission of diabetes in up to 70% of individuals) [75]. The sooner the patient implements elements of a healthy lifestyle, the better the results. In the look-AHEAD trial, which also demonstrated correlation between degree of weight loss and DM remission, the likelihood of remission was inversely correlated with the duration of diabetes [76].

### 2.2. RAAS Blockade

Despite significant progress in pharmacological nephroprotection, angiotensin-converting enzyme inhibitors (ACEi) or angiotensin receptor blockers (ARB) remain the cornerstone treatment of ORG. It is not surprising since over-activation of RAAS plays a central role in pathogenesis and progression of ORG. The effectiveness of RAAS blockade is undisputable among patients with CKD regardless of etiology. Interestingly, these drugs actually may be even more effective in obese patients than in those with normal BMI. In the Ramipril Efficacy In Nephropathy (REIN) trial among 337 participants, 105 (31.1%) were overweight and 49 (14.5%) were obese. In post hoc analysis, tge effect of ramipril on renal events (defined as ESRD or doubling of serum creatinine) and reduction in proteinuria was more pronounced in obese as compared to the overweight patient (86% decrease for ESRD and 79% for the combined endpoint versus 45 and 48%, respectively) and overweighted compared to those with normal body mass (incidence rate reduction of 42 and 45%, respectively) [77]. Although cited data are very optimistic, effect of RAAS blockade in obese patients but without proteinuria remains uncertain in terms of reducing the rate of GFR loss [78].

Obesity is also associated with excessive adipocyte-derived aldosterone secretion, independent of the classical renin–angiotensin–aldosterone cascade [79,80]. Adding the mineralocorticoid receptor antagonist (MRA)—spironolactone—to the RAAS blocker may result in a significant reduction in proteinuria, without increasing the risk of hyperkalemia [81]. Another drug in nephrologist armamentarium is finerenone. The FIDELIO-DKD trial proved that in patients with CKD and type 2 diabetes treatment with finerenone lowered the risks of CKD progression (and also number of cardiovascular events). All end-points defined in this trail were independent from presence and advancement of obesity [82].

### 2.3. Sodium-Glucose Cotransporter 2 Inhibitors (SGLT2i)

SGLT2i are undeniable breakthrough in renoprotection. Several trials convince that these drugs reduce the risk of renal adverse events [83,84,85]. Of importance, SGLT2i may be particularly beneficial for patients with ORG. First, they promote weight loss through excreting approximately 60–100 g of glucose per day with urine allowing for small but significant and sustained weight loss [86,87]. Second, they can reduce accumulation of visceral adipose tissue. Empagliflozin was proven to significantly reduce peri-hepatic fat and reduce ALT activity in patients with nonalcoholic fatty liver disease (NAFLD) [88]. Dapagliflozin compared to placebo showed statistically significant reduction in waist circumference, total body fat mass, visceral adipose tissue, and subcutaneous adipose tissue deposits [87]. Canagliflozin reduces epicardial fat [89]. SGLT2i also reduce serum leptin, increase adiponectin, and reduce insulin resistance [90].

### 2.4. GLP-1 Receptor Agonists

Following the undeniable success of SGLT2 inhibitorsm we witnessed another breakthrough, FDA-approved liraglutide and semaglutide for weight loss. In a double-blind trial, which enrolled 1961 overweight or obese adults, participants were randomized to once-weekly subcutaneous semaglutide (at the maximum dose of 2.4 mg) or placebo, plus lifestyle intervention. The results were spectacular. There was a change in body weight from baseline to week 68 was 15.3 kg in the semaglutide group as compared to 2.6 kg in the placebo group. In the series of STEP trials (STEP 1–3) semaglutide was demonstrated to reduce baseline urine albumin–creatinine ratio (UACR) without significant effect on eGFR [91,92]. The nephroprotective and life-saving properties of subcutaneous once-weekly semaglutide in patients with established and advanced CKD were demonstrated in the FLOW trial. In this trial, 3533 participants with type 2 diabetes and chronic kidney disease were included and randomized to semaglutide and the placebo (with standard of care). The primary outcome was major kidney disease events, a composite of the onset of kidney failure (dialysis, transplantation, or an eGFR of <15 mL per minute per 1.73 m^2^), at least a 50% reduction in the eGFR from baseline, or death from kidney-related or cardiovascular causes. Even more importantly, semaglutide significantly reduced the all-cause mortality in this high-risk group of patients. The trial was stopped after interim analysis because the risk of a primary outcome event was 24% lower in the semaglutide group than in the placebo [93]. Also, post hoc analysis of SUSTAIN 6 and PIONEER 6 showed clinically meaningful reduction in risk of chronic kidney disease regardless of HbA1c, BP, BMI, and UACR levels [94]. The SELECT trial that included obese patients without diabetes, but having high prevalence of cardiovascular and/or cerebrovascular disease randomized to subcutaneous once-weekly semaglutide or placebo demonstrated that semaglutide decreased primary cardiovascular composite end-point by 20% and heart failure composite end-point by 18%, and it prolonged life of those high risk patients (reduction in risk of death for any cause by 19% [95]. The onset of composite renal end-points that included renal death, the need of renal replacement therapy, decrease in GFR below 15 mL/min./1.73 m^2^, decrease in GFR by ≥50% from baseline, or de novo persistent macroalbuminuria, was also significantly reduced (22% reduction) in patients on semaglutide. This reduction was mainly driven by a significantly lower incidence of macroalbuminuria in semaglutide-treated patients. The rate of GFR reduction was lower in patients receiving semaglutide by statistically significant and clinically meaningful value of 0.39 mL/min./1.73 m^2^ per year, being slower by 2.19 mL/min./1.73 m^2^ per year among patients with baseline eGFR < 60 mL/min./1.73 m^2^. It must be kept in mind while interpreting these results that the mean baseline eGFR was normal in the whole study cohort (mean 82.4 ± 17.5 mL/min./1.73 m^2^) with a low percentage of patients with CKD stage 3 (i.e., eGFR < 60 mL/min./1.73 m^2^) [96].

One year after approval of subcutaneous semaglutide, another milestone therapy has been introduced. The FDA approved the use of tirzepatide for obesity treatment. It is the novel drug, first-in-class once-weekly subcutaneous gastric inhibitory polypeptide analog and glucagon-like peptide-1 receptor. The SURMOUNT-1 trial (Tirzepatide for Weight Reduction in Chinese Adults With Obesity) recruited 2539 participants with obesity, of whom 1032 also had prediabetes. Mean percent change in body weight at week 52 was −13.6% and −17.5% with tirzepatide 10 mg and 15 mg, respectively, compared with −2.3% with the placebo [97]. The drug also demonstrated positive results in the spectrum of obesity-related diseases like hypertension, steatohepatitis with liver fibrosis, or obstructive sleep apnea [98,99]. Randomized control trials are necessary to determine its effect in patients with CKD, although meta analysis of 15 RCTs suggests that tirzepatide positively impacts UACR without detrimental effects on eGFR in subjects with T2D and obesity without T2D [100]. The series of SURPASS trials performed in T2D patients, with significant proportion of overweight or obese (SURPASS-1, tirzepatide vs. placebo, SURPASS-2, tirzepatide vs. subcutaneous semaglutide, SURPASS-3, tirzepatide vs. insulin degludec, SURPASS-4, tirzepatide vs. insulin glargine, SURPASS-5, tirzepatide vs. placebo; in each trial placebo, active comparator or tirzepatide was added to different background therapies) was a subject of renal end-point-oriented meta-analysis. Follow-ups in the SURPASS trials lasted 40–42 weeks, and 5, 10, and 15 mg weekly doses were analyzed. The drug significantly reduced UACR, and the reduction was dose-dependent (−19.3% for 5 mg, −22% for 10 mg, and −26.3% for 15 mg). UACR reduction following tirzepatide was independent from background treatment with SGLT2i or drugs inhibiting the renin–angiotensin–aldosterone system. In a trial directly comparing tirzepatide and semaglutide (SURPASS-2), the rate of UACR reduction was equal for both drugs. Tirzepatide had a neutral effect on eGFR. Interpretation of the impact of tirzepatide on renal end-points needs caution since patients included in the SURPASS trials had normal kidney function (median UACR 11 mg/g, less than 7% with UACR > 300 mg/g mean eGFR ranging between 89 and 97 mL/min./1.73 m^2^ with only 5–8% of patients with eGFR < 60 mL/min./1.73 m^2^ [101]. In contrast to semaglutide, outcome trial results (analyzing cardiovascular and renal end-points in high-risk patients) are not yet available. An overview of the most important clinical trials is presented in Table 1.

### 2.5. Cagrilintide

Cagrilintide is a long-acting amylin analog. Amylin, released with insulin from beta cells in the pancreas, induces a satiating effect via both the homoeostatic and hedonic regions of the brain. The trial tested the hypothesis that cagrilintide added to semaglutide as a standard of care vs. semaglutide plus placebo would be more effective in metabolic control of diabetes and body mass reduction in diabetic patients. It has been demonstrated in this randomized clinical trial that treatment with cagrilintide added to semagluted resulted in relevant improvement in glycemic control and significantly greater weight loss when compared to semaglutide alone. Cagrilintide was also well tolerated [103]. There is an ongoing, actively recruiting clinical trial with combination of semaglutide and cagrilintide investigating if kidney damage in obese patients with CKD and type 2 diabetes can be reduced by this combination of drugs (NCT06131372).

### 2.6. Gut Microbiota Manipulation

Gut microbiota have been identified as an important player in regulation of metabolism and—when abnormal—as a contributor to metabolic disorders. For these reasons, modifying gut microbiota has been identified as a promising approach in the treatment of obesity-related conditions. Gut microbiota is a subject of substantial quantitative and qualitative abnormalities in CKD and end stage kidney disease; thus, it became an obvious object of interest [104].

It was proven in animal model, that gut microbiota plays a role in weight gain. Transplantation of microbiota from obese mice to germ-free mice led to 60% increase in body fat content and insulin resistance within 14 days, despite reduced food intake [105].

Recently, the role of prebiotics (non-digestible food ingredients that selectively stimulate the growth and activity of a limited number of bacteria in the digestive tract), probiotics (beneficial microorganisms), and synbiotics (combination of two mentioned above) may become the treatment options for obese patients with diabetes mellitus. Supplementation of *Bifidobacterium lactis* and *Lactobacillus rhamnosus* was proven to upregulate PPARγ and lipoprotein lipase expression and to enhance insulin sensitivity and triglycerides clearance [106]. *Bifidobacterium longum* and *Ligilactobacillus salivarius* upregulates GLP-1 expression [107]. Probiotics, prebiotics, synbiotics, and postbiotics (PPSP) are beneficial for weight loss, improving blood lipids and inflammatory markers such as TNF-α and IL-6, decreasing HbA1c level [108]. As for today, data on the impact of gut microbiota manipulation on ORG are lacking.

### 2.7. Melatonin

Melatonin is a pleiotropic indoleamine hormone produced mostly by the pineal gland. Several animal studies have shown that it modulates lipid metabolism, and it can significantly reduce the levels of serum triglyceride, total cholesterol, and low-density lipoprotein-cholesterol in rodents fed by high-fat diet [109]. Melatonin has been confirmed to improve insulin sensitivity, induce β-cell regeneration in the pancreas, and promote hepatic glycogen synthesis, thus reducing hyperglycemia in rodents [110]. Higher levels of endogenous nocturnal melatonin secretion are negatively related to the insulin level and onset of insulin resistance [111]. It has been reported that melatonin can regulate levels of leptin and adiponectin, fat deposition, brown adipose tissue growth, adipogenesis, and white adipose tissue browning, which in turn affects energy consumption. Because of its antioxidant and anti-inflammatory effects, melatonin has been investigated in different types of kidney injury. Positive effects of melatonin supplementation were proven in lupus nephritis, membranous nephropathy, contrast-induced acute kidney injury, or drug-related AKI [112,113,114,115,116]. It was also beneficial in patients with diabetes [117]. In numerous studies on animal models (mostly in Zucker Diabetic Fatty rats), melatonin improves kidney function by reducing urine albumin/creatinine ratio, BUN, serum creatinine level and also kidney morphology by limiting basement membrane thickening, inflammatory cell infiltration, vacuolization in tubular cells, and deposition of extracellular matrix [118]. All of the above make melatonin a possible therapeutic option.

## 3. Conclusions

The kidneys belong to several victims of obesity. CKD developing as a consequence of obesity turns into the risk factor of accelerated atherogenic cardiovascular morbidity and heart failure. Both increasing albuminuria and decreasing GFR are well established and independent risk factors for adverse outcome and mortality in obese patients. Modern interventions (including bariatric surgery and lifestyle changes and pharmacological treatment) can be effective in weight reduction but also in kidney protection. The GLP1 receptor agonist semaglutide remains the key pharmacological intervention protecting kidneys and saving lives in obese patients with diabetes and established CKD. Both semaglutide and the GLP1/GIP receptor agonist tirzepatide demonstrated several nephroprotective properties in patients with obesity regardless of diabetes.

## Figures and Tables

**Table 1:** Comparison of renal effects of tirzepatide and semaglutide in obese patients.

Name of the Trial	Study Drug and Dose	N	Concomitant Therapy	Comparator	BMI [kg/m^2^]	BMI ≥ 35 kg/m^2^	Reduction in UACR [%]
SURPASS-1 [101,102]	Tirzepatide 5 mg 10 mg 15 mg	478	diet and exercise	placebo	31.9 (6.6)	131 (27.4%)	−43.4 −43 −31.3
SURPASS-2 [101,102]	Tirzepatide 5 mg 10 mg 15 mg	1878	metformin	semaglutide 1 mg	34.2 (6.9)	690 (36.7%)	−4.8 2.8 −11.1
SURPASS-3 [101,102]	Tirzepatide 5 mg 10 mg 15 mg	1437	metformin with or without SGLT2i	insulin degludec	33.5 (6.1)	495 (34.4%)	−12.7 −14.9 −23.3
SURPASS-4 [101,102]	Tirzepatide 5 mg 10 mg 15 mg	1995	metformin or sulfonylurea or SGLT2i	insulin glargine	32.6 (5.5)	556 (27.9%)	−23.3 −32.3 −35.2
SURPASS-5 [101,102]	Tirzepatide 5 mg 10 mg 15 mg	475	insulin glargine with or without metformin	placebo	33.4 (6.1)	180 (37.9%)	−10.7 −33.8 −29.4
SURMOUNT-1 [102]	Tirzepatide 5 mg 10 mg 15 mg	1032		placebo	38.8 (7.10)	674 (65.3%)	6.2 −9.5 −13.9
FLOW [93,102]	Semaglutide	3533	RAASi with or without SGLT2i	placebo	32.0 (6.3)	957 (27%)	−40
SELECT [96]	Semaglutide 2.4 mg	17,605	local clinical practice of CV risk factors management	placebo	33.34 (5.04)	5106 (29%)	−10.7
STEP 1 [91]	semaglutide 2.4 mg	1961	lifestyle intervention	placebo	37.9 (6.6)	1201 (61.2%)	No data available
STEP 2 [91]	semaglutide 1 mg 2.4 mg	1210	lifestyle intervention	placebo	35.7 (6.3)	561 (46.3%)	−14.8 −20.6
STEP 3 [91]	semaglutide 2.4 mg	611	intensive behavioral therapy	placebo	38.0 (6.7)	389 (63.7%)	No data available

## Data Availability

Data sharing is not applicable.

## References

[1] Ogden C.L., Carroll M.D., Kit B.K., Flegal K.M. (2014). Prevalence of childhood and adult obesity in the United States, 2011–2012. JAMA.

[2] Afshin A., Forouzanfar M.H., Reitsma M.B., Sur P., Estep K., Lee A., Marczak L., Mokdad A.H., Moradi-Lakeh M., GBD 2015 Obesity Collaborators (2017). Health Effects of Overweight and Obesity in 195 Countries over 25 Years. N. Engl. J. Med..

[3] Lobstein T., Brinsden H. (2019). Atlas of Childhood Obesity.

[4] Godfrey K.M., Reynolds R.M., Prescott S.L., Nyirenda M., Jaddoe V.W., Eriksson J.G., Broekman B.F. (2017). Influence of maternal obesity on the long-term health of offspring. Lancet Diabetes Endocrinol..

[5] Kambham N., Markowitz G.S., Valeri A.M., Lin J., D’Agati V.D. (2001). Obesity-related glomerulopathy: An emerging epidemic. Kidney Int..

[6] Chander P.N., Gealekman O., Brodsky S.V., Elitok S., Tojo A., Crabtree M., Gross S.S., Goligorsky M.S. (2004). Nephropathy in Zucker diabetic fat rat is associated with oxidative and nitrosative stress: Prevention by chronic therapy with a peroxynitrite scavenger ebselen. J. Am. Soc. Nephrol..

[7] Serra A., Romero R., Lopez D., Navarro M., Esteve A., Perez N., Alastrue A., Ariza A. (2008). Renal injury in the extremely obese patients with normal renal function. Kidney Int..

[8] Foster M.C., Hwang S.J., Larson M.G., Lichtman J.H., Parikh N.I., Vasan R.S., Levy D., Fox C.S. (2008). Overweight, obesity, and the development of stage 3 CKD: The Framingham Heart Study. Am. J. Kidney Dis..

[9] Pinto-Sietsma S.J., Navis G., Janssen W.M., de Zeeuw D., Gans R.O., de Jong P.E., PREVEND Study Group (2003). A central body fat distribution is related to renal function impairment, even in lean subjects. Am. J. Kidney Dis..

[10] Cohen A.H. (1975). Massive obesity and the kidney. A morphologic and statistical study. Am. J. Pathol..

[11] Chen H.M., Liu Z.H., Zeng C.H., Li S.J., Wang Q.W., Li L.S. (2006). Podocyte lesions in patients with obesity-related glomerulopathy. Am. J. Kidney Dis..

[12] Praga M., Hernández E., Morales E., Campos A.P., Valero M.A., Martínez M.A., León M. (2001). Clinical features and long-term outcome of obesity-associated focal segmental glomerulosclerosis. Nephrol. Dial. Transplant..

[13] Darouich S., Goucha R., Jaafoura M.H., Zekri S., Ben Maiz H., Kheder A. (2011). Clinicopathological characteristics of obesity-associated focal segmental glomerulosclerosis. Ultrastruct. Pathol..

[14] Chagnac A., Weinstein T., Korzets A., Ramadan E., Hirsch J., Gafter U. (2000). Glomerular hemodynamics in severe obesity. Am. J. Physiol. Renal Physiol..

[15] Goumenos D.S., Kawar B., El Nahas M., Conti S., Wagner B., Spyropoulos C., Vlachojannis J.G., Benigni A., Kalfarentzos F. (2009). Early histological changes in the kidney of people with morbid obesity. Nephrol. Dial. Transplant..

[16] Bobulescu I.A., Lotan Y., Zhang J., Rosenthal T.R., Rogers J.T., Adams-Huet B., Sakhaee K., Moe O.W. (2014). Triglycerides in the human kidney cortex: Relationship with body size. PLoS ONE.

[17] Cao Y. (2010). Adipose tissue angiogenesis as a therapeutic target for obesity and metabolic diseases. Nat. Rev. Drug Discov..

[18] Berfield A.K., Andress D.L., Abrass C.K. (2002). IGF-1-induced lipid accumulation impairs mesangial cell migration and contractile function. Kidney Int..

[19] van Zonneveld A.J., Rabelink T.J. (2001). Mesangial cells defy LDL receptor paradigm. Kidney Int..

[20] Coimbra T.M., Janssen U., Gröne H.J., Ostendorf T., Kunter U., Schmidt H., Brabant G., Floege J. (2000). Early events leading to renal injury in obese Zucker (fatty) rats with type II diabetes. Kidney Int..

[21] Jalovaara K., Santaniemi M., Timonen M., Jokelainen J., Kesäniemi Y.A., Ukkola O., Keinänen-Kiukaanniemi S., Rajala U. (2008). Low serum adiponectin level as a predictor of impaired glucose regulation and type 2 diabetes mellitus in a middle-aged Finnish population. Metabolism.

[22] Johnson D.W., Armstrong K., Campbell S.B., Mudge D.W., Hawley C.M., Coombes J.S., Prins J.B., Isbel N.M. (2007). Metabolic syndrome in severe chronic kidney disease: Prevalence, predictors, prognostic significance and effects of risk factor modification. Nephrology.

[23] Ohashi K., Iwatani H., Kihara S., Nakagawa Y., Komura N., Fujita K., Maeda N., Nishida M., Katsube F., Shimomura I. (2007). Exacerbation of albuminuria and renal fibrosis in subtotal renal ablation model of adiponectin-knockout mice. Arterioscler. Thromb. Vasc. Biol..

[24] Yamauchi T., Kamon J., Waki H., Terauchi Y., Kubota N., Hara K., Mori Y., Ide T., Murakami K., Tsuboyama-Kasaoka N. (2001). The fat-derived hormone adiponectin reverses insulin resistance associated with both lipoatrophy and obesity. Nat. Med..

[25] Kadowaki T., Yamauchi T., Kubota N., Hara K., Ueki K., Tobe K. (2006). Adiponectin and adiponectin receptors in insulin resistance, diabetes, and the metabolic syndrome. J. Clin. Invest..

[26] Meyvis K., Verrijken A., Wouters K., Van Gaal L. (2013). Plasma adiponectin level is inversely correlated with albuminuria in overweight and obese nondiabetic individuals. Metabolism.

[27] Choi H.M., Doss H.M., Kim K.S. (2020). Multifaceted Physiological Roles of Adiponectin in Inflammation and Diseases. Int. J. Mol. Sci..

[28] Rovin B.H., Song H. (2006). Chemokine induction by the adipocyte-derived cytokine adiponectin. Clin. Immunol..

[29] Song S.H., Oh T.R., Choi H.S., Kim C.S., Ma S.K., Oh K.H., Ahn C., Kim S.W., Bae E.H. (2020). High serum adiponectin as a biomarker of renal dysfunction: Results from the KNOW-CKD study. Sci. Rep..

[30] Sharma K., Ramachandrarao S., Qiu G., Usui H.K., Zhu Y., Dunn S.R., Ouedraogo R., Hough K., McCue P., Chan L. (2008). Adiponectin regulates albuminuria and podocyte function in mice. J. Clin. Invest..

[31] Rutkowski J.M., Wang Z.V., Park A.S., Zhang J., Zhang D., Hu M.C., Moe O.W., Susztak K., Scherer P.E. (2013). Adiponectin promotes functional recovery after podocyte ablation. J. Am. Soc. Nephrol..

[32] Su Y.X., Deng H.C., Zhang M.X., Long J., Peng Z.G. (2012). Adiponectin inhibits PDGF-induced mesangial cell proliferation: Regulation of mammalian target of rapamycin-mediated survival pathway by adenosine 5-monophosphate-activated protein kinase. Horm. Metab. Res..

[33] Zhang F., Chen Y., Heiman M., Dimarchi R. (2005). Leptin: Structure, function and biology. Vitam. Horm..

[34] Teixeira T.M., da Costa D.C., Resende A.C., Soulage C.O., Bezerra F.F., Daleprane J.B. (2017). Activation of Nrf2-Antioxidant Signaling by 1,25-Dihydroxycholecalciferol Prevents Leptin-Induced Oxidative Stress and Inflammation in Human Endothelial Cells. J. Nutr..

[35] Considine R.V. (2005). Human leptin: An adipocyte hormone with weight-regulatory and endocrine functions. Semin. Vasc. Med..

[36] Han D.C., Isono M., Chen S., Casaretto A., Hong S.W., Wolf G., Ziyadeh F.N. (2001). Leptin stimulates type I collagen production in db/db mesangial cells: Glucose uptake and TGF-beta type II receptor expression. Kidney Int..

[37] Ruiz-Ortega M., Rayego-Mateos S., Lamas S., Ortiz A., Rodrigues-Diez R.R. (2020). Targeting the progression of chronic kidney disease. Nat. Rev. Nephrol..

[38] Declèves A.E., Sharma K. (2015). Obesity and kidney disease: Differential effects of obesity on adipose tissue and kidney inflammation and fibrosis. Curr. Opin. Nephrol. Hypertens..

[39] Coward R., Fornoni A. (2015). Insulin signaling: Implications for podocyte biology in diabetic kidney disease. Curr. Opin. Nephrol. Hypertens..

[40] Lin L., Tan W., Pan X., Tian E., Wu Z., Yang J. (2022). Metabolic Syndrome-Related Kidney Injury: A Review and Update. Front. Endocrinol..

[41] Tsuruta H., Yasuda-Yamahara M., Yoshibayashi M., Kuwagata S., Yamahara K., Tanaka-Sasaki Y., Chin-Kanasaki M., Matsumoto S., Ema M., Kume S. (2024). Fructose overconsumption accelerates renal dysfunction with aberrant glomerular endothelial-mesangial cell interactions in db/db mice. Biochim. Biophys. Acta Mol. Basis Dis..

[42] Feng Z., Zhu L., Wu J. (2020). RAGE signalling in obesity and diabetes: Focus on the adipose tissue macrophage. Adipocyte.

[43] Machado-Lima A., López-Díez R., Iborra R.T., Pinto R.S., Daffu G., Shen X., Nakandakare E.R., Machado U.F., Corrêa-Giannella M.L.C., Schmidt A.M. (2020). RAGE Mediates Cholesterol Efflux Impairment in Macrophages Caused by Human Advanced Glycated Albumin. Int. J. Mol. Sci..

[44] Guan Y., Wei X., Li J., Zhu Y., Luo P., Luo M. (2024). Obesity-related glomerulopathy: Recent advances in inflammatory mechanisms and related treatments. J. Leukoc. Biol..

[45] Sharma R., Gaze D.C., Pellerin D., Mehta R.L., Gregson H., Streather C.P., Collinson P.O., Brecker S.J. (2006). Ischemia-modified albumin predicts mortality in ESRD. Am. J. Kidney Dis..

[46] Endlich N., Kress K.R., Reiser J., Uttenweiler D., Kriz W., Mundel P., Endlich K. (2001). Podocytes respond to mechanical stress in vitro. J. Am. Soc. Nephrol..

[47] Pecly I.M., Genelhu V., Francischetti E.A. (2006). Renal functional reserve in obesity hypertension. Int. J. Clin. Pract..

[48] Cortinovis M., Perico N., Ruggenenti P., Remuzzi A., Remuzzi G. (2022). Glomerular hyperfiltration. Nat. Rev. Nephrol..

[49] Ehrhart-Bornstein M., Arakelyan K., Krug A.W., Scherbaum W.A., Bornstein S.R. (2004). Fat cells may be the obesity-hypertension link: Human adipogenic factors stimulate aldosterone secretion from adrenocortical cells. Endocr. Res..

[50] Kennedy C.R., Burns K.D. (2001). Angiotensin II as a mediator of renal tubular transport. Contrib. Nephrol..

[51] Davy K.P., Orr J.S. (2009). Sympathetic nervous system behavior in human obesity. Neurosci. Biobehav. Rev..

[52] Young C.N., Morgan D.A., Butler S.D., Mark A.L., Davisson R.L. (2013). The brain subfornical organ mediates leptin-induced increases in renal sympathetic activity but not its metabolic effects. Hypertension.

[53] Vallon V., Blantz R.C., Thomson S. (2003). Glomerular hyperfiltration and the salt paradox in early [corrected] type 1 diabetes mellitus: A tubulo-centric view. J. Am. Soc. Nephrol..

[54] Ott C.E., Haas J.A., Cuche J.L., Knox F.G. (1975). Effect of increased peritubule protein concentration on proximal tubule reabsorption in the presence and absence of extracellular volume expansion. J. Clin. Invest..

[55] Kriz W., Lemley K.V. (2015). A potential role for mechanical forces in the detachment of podocytes and the progression of CKD. J. Am. Soc. Nephrol..

[56] Huang C., Bruggeman L.A., Hydo L.M., Miller R.T. (2012). Shear stress induces cell apoptosis via a c-Src-phospholipase D-mTOR signaling pathway in cultured podocytes. Exp. Cell Res..

[57] Srivastava T., Dai H., Heruth D.P., Alon U.S., Garola R.E., Zhou J., Duncan R.S., El-Meanawy A., McCarthy E.T., Sharma R. (2018). Mechanotransduction signaling in podocytes from fluid flow shear stress. Am. J. Physiol. Renal Physiol..

[58] Srivastava T., Celsi G.E., Sharma M., Dai H., McCarthy E.T., Ruiz M., Cudmore P.A., Alon U.S., Sharma R., Savin V.A. (2014). Fluid flow shear stress over podocytes is increased in the solitary kidney. Nephrol. Dial. Transplant..

[59] Manno C., Campobasso N., Nardecchia A., Triggiani V., Zupo R., Gesualdo L., Silvestris F., De Pergola G. (2019). Relationship of para- and perirenal fat and epicardial fat with metabolic parameters in overweight and obese subjects. Eat. Weight. Disord..

[60] GBD 2021 Stroke Risk Factor Collaborators (2024). Global, regional, and national burden of stroke and its risk factors, 1990–2021, a systematic analysis for the Global Burden of Disease Study 2021. Lancet Neurol..

[61] Liu B.X., Sun W., Kong X.Q. (2019). Perirenal Fat: A Unique Fat Pad and Potential Target for Cardiovascular Disease. Angiology.

[62] Huang N., Mao E.W., Hou N.N., Liu Y.P., Han F., Sun X.D. (2020). Novel insight into perirenal adipose tissue: A neglected adipose depot linking cardiovascular and chronic kidney disease. World J. Diabetes.

[63] Choi J.W., Lee C.M., Kang B.K., Kim M. (2024). Perirenal fat thickness is an independent predictor for metabolic syndrome in steatotic liver disease. Sci. Rep..

[64] Sun X., Yu Y., Han L. (2013). High FFA levels related to microalbuminuria and uncoupling of VEGF-NO axis in obese rats. Int. Urol. Nephrol..

[65] Liu Y., Wang L., Luo M., Chen N., Deng X., He J., Zhang L., Luo P., Wu J. (2019). Inhibition of PAI-1 attenuates perirenal fat inflammation and the associated nephropathy in high-fat diet-induced obese mice. Am. J. Physiol. Endocrinol. Metab..

[66] Grassi G., Biffi A., Seravalle G., Trevano F.Q., Dell’Oro R., Corrao G., Mancia G. (2019). Sympathetic Neural Overdrive in the Obese and Overweight State. Hypertension.

[67] Cao Q., Zhang J., Yu Q., Wang J., Dai M., Zhang Y., Luo Q., Bao M. (2019). Carotid baroreceptor stimulation in obese rats affects white and brown adipose tissues differently in metabolic protection. J. Lipid Res..

[68] Chen X., Qin Y., Hu J., Shen Y., Mao Y., Xie L., Li J., Wang J., Yang S., Li Q. (2024). Perirenal fat and chronic kidney disease in type 2 diabetes: The mediation role of afferent arteriolar resistance. Diabetes Metab..

[69] Hu H., Liang W., Zhang Z., Liu Z., Chu F., Bao Y., Ran J., Ding G. (2022). The Utility of Perirenal Fat in Determining the Risk of Onset and Progression of Diabetic Kidney Disease. Int. J. Endocrinol..

[70] Hu H., Zhang Z., Liu Z., Chu F., Ran J., Liang W. (2023). Thickened Perirenal Fat Predicts Poor Renal Outcome in Patients with Immunoglobulin A Nephropathy: A Population-Based Retrospective Cohort Study. Kidney Dis..

[71] Navaneethan S.D., Yehnert H., Moustarah F., Schreiber M.J., Schauer P.R., Beddhu S. (2009). Weight loss interventions in chronic kidney disease: A systematic review and meta-analysis. Clin. J. Am. Soc. Nephrol..

[72] Shulman A., Peltonen M., Sjöström C.D., Andersson-Assarsson J.C., Taube M., Sjöholm K., le Roux C.W., Carlsson L.M.S., Svensson P.A. (2018). Incidence of end-stage renal disease following bariatric surgery in the Swedish Obese Subjects Study. Int. J. Obes..

[73] Conley M.M., McFarlane C.M., Johnson D.W., Kelly J.T., Campbell K.L., MacLaughlin H.L. (2021). Interventions for weight loss in people with chronic kidney disease who are overweight or obese. Cochrane Database Syst. Rev..

[74] Chang S.H., Stoll C.R., Song J., Varela J.E., Eagon C.J., Colditz G.A. (2014). The effectiveness and risks of bariatric surgery: An updated systematic review and meta-analysis, 2003–2012. JAMA Surg..

[75] Steven S., Taylor R. (2015). Restoring normoglycaemia by use of a very low calorie diet in long- and short-duration Type 2 diabetes. Diabet. Med..

[76] Gregg E.W., Chen H., Wagenknecht L.E., Clark J.M., Delahanty L.M., Bantle J., Pownall H.J., Johnson K.C., Safford M.M., Kitabchi A.E. (2012). Look AHEAD Research Group. Association of an intensive lifestyle intervention with remission of type 2 diabetes. JAMA.

[77] Mallamaci F., Ruggenenti P., Perna A., Leonardis D., Tripepi R., Tripepi G., Remuzzi G., Zoccali C. (2011). REIN Study Group. ACE inhibition is renoprotective among obese patients with proteinuria. J. Am. Soc. Nephrol..

[78] Tofte N., Lindhardt M., Adamova K., Bakker S.J.L., Beige J., Beulens J.W.J., Birkenfeld A.L., Currie G., Delles C., Dimos I. (2020). Early detection of diabetic kidney disease by urinary proteomics and subsequent intervention with spironolactone to delay progression (PRIORITY): A prospective observational study and embedded randomised placebo-controlled trial. Lancet Diabetes Endocrinol..

[79] Briones A.M., Nguyen Dinh Cat A., Callera G.E., Yogi A., Burger D., He Y., Corrêa J.W., Gagnon A.M., Gomez-Sanchez C.E., Gomez-Sanchez E.P. (2012). Adipocytes produce aldosterone through calcineurin-dependent signaling pathways: Implications in diabetes mellitus-associated obesity and vascular dysfunction. Hypertension.

[80] Rüster C., Wolf G. (2013). The role of the renin-angiotensin-aldosterone system in obesity-related renal diseases. Semin. Nephrol..

[81] Morales E., Gutiérrez E., Caro J., Sevillano A., Rojas-Rivera J., Praga M. (2015). Beneficial long-term effect of aldosterone antagonist added to a traditional blockade of the renin-angiotensin-aldosterone system among patients with obesity and proteinuria. Nefrologia.

[82] Bakris G.L., Agarwal R., Anker S.D., Pitt B., Ruilope L.M., Rossing P., Kolkhof P., Nowack C., Schloemer P., Joseph A. (2020). FIDELIO-DKD Investigators. Effect of Finerenone on Chronic Kidney Disease Outcomes in Type 2 Diabetes. N. Engl. J. Med..

[83] Perkovic V., Jardine M.J., Neal B., Bompoint S., Heerspink H.J.L., Charytan D.M., Edwards R., Agarwal R., Bakris G., Bull S. (2019). Canagliflozin and Renal Outcomes in Type 2 Diabetes and Nephropathy. N. Engl. J. Med..

[84] Herrington W.G., Staplin N., Wanner C., Green J.B., Hauske S.J., Emberson J.R., Preiss D., Judge P., Mayne K.J., The EMPA-KIDNEY Collaborative Group (2023). Empagliflozin in Patients with Chronic Kidney Disease. N. Engl. J. Med..

[85] Wheeler D.C., Stefánsson B.V., Jongs N., Chertow G.M., Greene T., Hou F.F., McMurray J.J.V., Correa-Rotter R., Rossing P., DAPA-CKD Trial Committees and Investigators (2021). Effects of dapagliflozin on major adverse kidney and cardiovascular events in patients with diabetic and non-diabetic chronic kidney disease: A prespecified analysis from the DAPA-CKD trial. Lancet Diabetes Endocrinol..

[86] Pereira M.J., Eriksson J.W. (2019). Emerging Role of SGLT-2 Inhibitors for the Treatment of Obesity. Drugs.

[87] Bolinder J., Ljunggren Ö., Kullberg J., Johansson L., Wilding J., Langkilde A.M., Sugg J., Parikh S. (2012). Effects of dapagliflozin on body weight, total fat mass, and regional adipose tissue distribution in patients with type 2 diabetes mellitus with inadequate glycemic control on metformin. J. Clin. Endocrinol. Metab..

[88] Kuchay M.S., Krishan S., Mishra S.K., Farooqui K.J., Singh M.K., Wasir J.S., Bansal B., Kaur P., Jevalikar G., Gill H.K. (2018). Effect of Empagliflozin on Liver Fat in Patients With Type 2 Diabetes and Nonalcoholic Fatty Liver Disease: A Randomized Controlled Trial (E-LIFT Trial). Diabetes Care.

[89] Yagi S., Hirata Y., Ise T., Kusunose K., Yamada H., Fukuda D., Salim H.M., Maimaituxun G., Nishio S., Takagawa Y. (2017). Canagliflozin reduces epicardial fat in patients with type 2 diabetes mellitus. Diabetol. Metab. Syndr..

[90] Wu P., Wen W., Li J., Xu J., Zhao M., Chen H., Sun J. (2019). Systematic Review and Meta-Analysis of Randomized Controlled Trials on the Effect of SGLT2 Inhibitor on Blood Leptin and Adiponectin Level in Patients with Type 2 Diabetes. Horm. Metab. Res..

[91] Heerspink H.J.L., Apperloo E., Davies M., Dicker D., Kandler K., Rosenstock J., Sørrig R., Lawson J., Zeuthen N., Cherney D. (2023). Effects of Semaglutide on Albuminuria and Kidney Function in People With Overweight or Obesity With or Without Type 2 Diabetes: Exploratory Analysis From the STEP 1, 2, and 3 Trials. Diabetes Care.

[92] Wilding J.P.H., Batterham R.L., Calanna S., Davies M., Van Gaal L.F., Lingvay I., McGowan B.M., Rosenstock J., Tran M.T.D., Wadden T.A. (2021). STEP 1 Study Group. Once-Weekly Semaglutide in Adults with Overweight or Obesity. N. Engl. J. Med..

[93] Perkovic V., Tuttle K.R., Rossing P., Mahaffey K.W., Mann J.F.E., Bakris G., Baeres F.M.M., Idorn T., Bosch-Traberg H., FLOW Trial Committees and Investigators (2024). Effects of Semaglutide on Chronic Kidney Disease in Patients with Type 2 Diabetes. N. Engl. J. Med..

[94] Apperloo E.M., Cherney D.Z.I., Kuhlman A.B., Mann J.F.E., Rasmussen S., Rossing P., Tuttle K.R., Vrhnjak B., Heerspink H.J.L. (2025). Effect of semaglutide on kidney function across different levels of baseline HbA1c, blood pressure, body weight and albuminuria in SUSTAIN 6 and PIONEER 6. Nephrol. Dial. Transplant..

[95] Lincoff A.M., Brown-Frandsen K., Colhoun H.M., Deanfield J., Emerson S.S., Esbjerg S., Hardt-Lindberg S., Hovingh G.K., Kahn S.E., SELECT Trial Investigators (2023). Semaglutide and Cardiovascular Outcomes in Obesity without Diabetes. N. Engl. J. Med..

[96] Colhoun H.M., Lingvay I., Brown P.M., Deanfield J., Brown-Frandsen K., Kahn S.E., Plutzky J., Node K., Parkhomenko A., Rydén L. (2024). Long-term kidney outcomes of semaglutide in obesity and cardiovascular disease in the SELECT trial. Nat. Med..

[97] Jastreboff A.M., le Roux C.W., Stefanski A., Aronne L.J., Halpern B., Wharton S., Wilding J.P.H., Perreault L., Zhang S., SURMOUNT-1 Investigators (2025). Tirzepatide for Obesity Treatment and Diabetes Prevention. N. Engl. J. Med..

[98] Loomba R., Hartman M.L., Lawitz E.J., Vuppalanchi R., Boursier J., Bugianesi E., Yoneda M., Behling C., Cummings O.W., SYNERGY-NASH Investigators (2024). Tirzepatide for Metabolic Dysfunction-Associated Steatohepatitis with Liver Fibrosis. N. Engl. J. Med..

[99] Malhotra A., Grunstein R.R., Fietze I., Weaver T.E., Redline S., Azarbarzin A., Sands S.A., Schwab R.J., Dunn J.P., SURMOUNT-OSA Investigators (2024). Tirzepatide for the Treatment of Obstructive Sleep Apnea and Obesity. N. Engl. J. Med..

[100] Kamrul-Hasan A., Patra S., Dutta D., Nagendra L., Muntahi-Reza A., Borozan S., Pappachan J.M. (2025). Renal effects and safety of tirzepatide in subjects with and without diabetes: A systematic review and meta-analysis. World J. Diabetes.

[101] Apperloo E.M., Tuttle K.R., Pavo I., Haupt A., Taylor R., Wiese R.J., Hemmingway A., Cherney D.Z.I., Sattar N., Heerspink H.J.L. (2025). Tirzepatide Associated With Reduced Albuminuria in Participants With Type 2 Diabetes: Pooled Post Hoc Analysis From the Randomized Active- and Placebo-Controlled SURPASS-1-5 Clinical Trials. Diabetes Care.

[102] Karakasis P., Patoulias D., Fragakis N., Klisic A., Rizzo M. (2024). Effect of tirzepatide on albuminuria levels and renal function in patients with type 2 diabetes mellitus: A systematic review and multilevel meta-analysis. Diabetes Obes. Metab..

[103] Frias J.P., Deenadayalan S., Erichsen L., Knop F.K., Lingvay I., Macura S., Mathieu C., Pedersen S.D., Davies M. (2023). Efficacy and safety of co-administered once-weekly cagrilintide 2·4 mg with once-weekly semaglutide 2·4 mg in type 2 diabetes: A multicentre, randomised, double-blind, active-controlled, phase 2 trial. Lancet.

[104] Popkov V.A., Zharikova A.A., Demchenko E.A., Andrianova N.V., Zorov D.B., Plotnikov E.Y. (2022). Gut Microbiota as a Source of Uremic Toxins. Int. J. Mol. Sci..

[105] Bäckhed F., Ding H., Wang T., Hooper L.V., Koh G.Y., Nagy A., Semenkovich C.F., Gordon J.I. (2004). The gut microbiota as an environmental factor that regulates fat storage. Proc. Natl. Acad. Sci. USA.

[106] Alard J., Lehrter V., Rhimi M., Mangin I., Peucelle V., Abraham A.L., Mariadassou M., Maguin E., Waligora-Dupriet A.J., Pot B. (2016). Beneficial metabolic effects of selected probiotics on diet-induced obesity and insulin resistance in mice are associated with improvement of dysbiotic gut microbiota. Environ. Microbiol..

[107] Alard J., Cudennec B., Boutillier D., Peucelle V., Descat A., Decoin R., Kuylle S., Jablaoui A., Rhimi M., Wolowczuk I. (2021). Multiple Selection Criteria for Probiotic Strains with High Potential for Obesity Management. Nutrients.

[108] Li H.Y., Zhou D.D., Gan R.Y., Huang S.Y., Zhao C.N., Shang A., Xu X.Y., Li H.B. (2021). Effects and Mechanisms of Probiotics, Prebiotics, Synbiotics, and Postbiotics on Metabolic Diseases Targeting Gut Microbiota: A Narrative Review. Nutrients.

[109] Ou T.H., Tung Y.T., Yang T.H., Chien Y.W. (2019). Melatonin Improves Fatty Liver Syndrome by Inhibiting the Lipogenesis Pathway in Hamsters with High-Fat Diet-Induced Hyperlipidemia. Nutrients.

[110] Ramirez A.V.G., Filho D.R., de Sá L.B.P.C. (2021). Melatonin and its Relationships with Diabetes and Obesity: A Literature Review. Curr. Diabetes Rev..

[111] McMullan C.J., Curhan G.C., Schernhammer E.S., Forman J.P. (2013). Association of nocturnal melatonin secretion with insulin resistance in nondiabetic young women. Am. J. Epidemiol..

[112] Bonomini F., Dos Santos M., Veronese F.V., Rezzani R. (2019). NLRP3 Inflammasome Modulation by Melatonin Supplementation in Chronic Pristane-Induced Lupus Nephritis. Int. J. Mol. Sci..

[113] Wu C.C., Lu K.C., Lin G.J., Hsieh H.Y., Chu P., Lin S.H., Sytwu H.K. (2012). Melatonin enhances endogenous heme oxygenase-1 and represses immune responses to ameliorate experimental murine membranous nephropathy. J. Pineal Res..

[114] Chalikias G., Drosos I., Tziakas D.N. (2016). Prevention of Contrast-Induced Acute Kidney Injury: An Update. Cardiovasc. Drugs Ther..

[115] Hong T.S., Briscese K., Yuan M., Deshpande K., Aleksunes L.M., Brunetti L. (2021). Renoprotective Effects of Melatonin against Vancomycin-Related Acute Kidney Injury in Hospitalized Patients: A Retrospective Cohort Study. Antimicrob. Agents Chemother..

[116] Kim J.W., Jo J., Kim J.Y., Choe M., Leem J., Park J.H. (2019). Melatonin Attenuates Cisplatin-Induced Acute Kidney Injury through Dual Suppression of Apoptosis and Necroptosis. Biology.

[117] Mok J.X., Ooi J.H., Ng K.Y., Koh R.Y., Chye S.M. (2019). A new prospective on the role of melatonin in diabetes and its complications. Horm. Mol. Biol. Clin. Investig..

[118] Promsan S., Lungkaphin A. (2020). The roles of melatonin on kidney injury in obese and diabetic conditions. Biofactors.

